# Effect of magnification factor by Galilean loupes on working posture of dental students in simulated clinical procedures: associations between direct and observational measurements

**DOI:** 10.7717/peerj.13021

**Published:** 2022-03-10

**Authors:** Júlia Margato Pazos, Simone Cecilio Hallak Regalo, Paulo de Vasconcelos, Juliana Alvares Duarte Bonini Campos, Patricia Petromilli Nordi Sasso Garcia

**Affiliations:** 1 Department of Social Dentistry, School of Dentistry of Araraquara, São Paulo State University (Unesp), Araraquara, São Paulo, Brazil; 2 Department of Basic and Oral Biology, School of Dentistry of Ribeirão Preto, University of São Paulo (USP), Ribeirão Preto, São Paulo, Brazil; 3 Department of Food and Nutrition, School of Pharmaceutical Sciences, São Paulo State Univeristy (Unesp), Araraquara, São Paulo, Brazil

**Keywords:** Visual acuity, Magnification, Occupational health, Ergonomics, Galilean loupe, Dentistry

## Abstract

**Objectives:**

To determine the effect of different levels of Galilean loupe magnification on working posture as measured by compliance with ergonomic posture positions, angular deviation from the neutral position of the neck, and muscle activation in the neck and upper back region during simulated clinical conditions.

**Methods:**

An experimental laboratory study was performed in which the dependent variables were compliance with ergonomic posture requirements while performing simulated restorative procedures in Restorative Dentistry, angular deviation from the neutral position of the neck, and muscle activation in the neck and upper back. The independent variable was the level of Galilean loupe magnification, which was tested at four levels (naked eye, 2.5× magnification, 3.0× magnification, and 3.5× magnification). The cavity preparations and Class I composite resin restorations were performed on artificial first molars on a mannequin in a dental chair. The Compliance Assessment of Dental Ergonomic Posture Requirements (CADEP) was used for the postural analysis; as was an analysis of the angular deviation from the neutral position of the neck and surface electromyography. Working posture was recorded on video over the course of the procedure. Participants were filmed from three different angles. The Compliance Assessment of Dental Ergonomic Posture Requirements (CADEP) assessed compliance with ergonomic posture requirements. A locally produced posture assessment software analyzed angular deviation. Surface electromyography bilaterally assessed activation of the sternocleidomastoid, descending trapezius and ascending trapezius muscles. Two-factor analysis of variance (ANOVA) and either Tukey’s post-hoc test or the Games-Howell post-hoc test were performed (α = 0.05).

**Results:**

During the cavity preparations and restorations, the use of Galilean loupes at all magnifications positively influenced working posture as measured by participants’ compliance with ergonomic posture positions (*p* < 0.01) and neck angulation (*p* < 0.01); the use of these loupes did not affect muscle activation in the regions evaluated (*p* > 0.05).

**Conclusion:**

The use of Galilean loupes had a positive effect on dental students’ working posture during the restoration procedures performed.

## Introduction

Musculoskeletal disorders are one of the most common occupational issues faced by dentists and may begin as early as the professional training period (Garcia & Campos, 2013; Corrocher et al., 2014; Onety et al., 2014). Various risk factors may contribute to the development of these disorders (Onety et al., 2014; Presoto & Garcia, 2016; Presoto, Wajngarten & Garcia, 2016); however, one of the major causes of musculoskeletal disorders is poor working posture (Presoto & Garcia, 2016; Valachi & Valachi, 2003; Biswas et al., 2012; Garcia, Wajngarten & Campos, 2018). In dental training period the small operating field and the high-precision movements required are the main causes of poor working posture (Garcia et al., 2017).

The use of loupes in restorative dentistry is a strategy that may be used to minimize this issue, since loupes improve visualization of the operating field (Garcia et al., 2017; Wajngarten & Garcia, 2016; Carpentier et al., 2019). There seems to be a consensus in the literature that the improvement in operating field visualization provided by loupes increases diagnostic performance and dental procedure quality, (Congdon, Tolle & Darby, 2012; Wajngarten & Garcia, 2019; Resende et al., 2008; Maillet et al., 2008; Maggio, Vilegas & Blatz, 2011; Forgie et al., 1999; Farook et al., 2013; Eichenberger et al., 2015; Wajngarten & Garcia, 2018) as well as the ability to maintain a neutral working posture (Wajngarten & Garcia, 2016; Congdon, Tolle & Darby, 2012; Wajngarten & Garcia, 2019; Resende et al., 2008).

Though the use of magnification has been highly recommended in the field of dentistry, there are few evidence-based studies (Corbella et al., 2018) regarding the effects of magnification on working posture with a broad postural analysis combining direct and observational methods.

Surface electromyography (EMG) is a direct method that may be combined with observational methods. It consists of a non-invasive method that directly assesses muscle contraction (Pazos & Garcia, 2020). A study combining observational and direct methods may be able to suggest the possible magnification levels that could improve dental surgeons’ working posture. It may also start the discussions about the implementation of this tool during the professional training period, (Eichenberger et al., 2018; Hermens et al., 2000) during which students are more receptive to ergonomic training (Carpentier et al., 2019).

Although there are several magnification systems, it was observed in a previous study that the Galilean system showed a positive effect in working posture, (Pazos et al., 2020) and may therefore be a good choice for loupe implementation during the training phase of dental education programs (Resende et al., 2008).

Thus, the aim of this study was to determine the effects of different levels of Galilean loupe magnification on working posture during restoration procedures in simulated clinical conditions.

## Materials and Methods

### Study design

This was an experimental laboratory study. The dependent variables consisted of compliance with ergonomic posture positions while performing simulated restoration procedures in a restorative dentistry course as evaluated by the Compliance Assessment of Dental Ergonomic Posture Requirements (CADEP), (Garcia, Wajngarten & Campos, 2018) angular deviation from a neutral neck position, and activation of the right and left sternocleidomastoid muscle, the right and left descending trapezius, and the right and left ascending trapezius muscles. The independent variables were the four levels of magnification (no magnification and Galilean loupes at 2.5×, 3.0×, and 3.5× magnification) and four levels of tooth (16: right maxillary first molar; 26: left maxillary first molar; 36: left mandibular first molar; and 46: right mandibular first molar).

The sample unit was the restorative procedure (cavity preparation and Class I composite resin restoration) of each artificial tooth and the minimal sample size was determined using data from a pilot study, a power of 80% and a significance level of 5%. This resulted in 20 restorative dental procedures for each experimental condition. Tooth and loupes were randomized so that 20 cavity preparations and 20 restorations of each tooth were carried out (16, 26, 36, 46) with each of the magnifying loupes (*n* = 80; N_cavity preparation_ = 320; N_restoration_ = 320).

This study was approved by the Research Ethics Committee of the School of Dentistry of São Paulo State University (UNESP), Araraquara (CAAE Registry No. 16453219.8.0000.5416). A written informed consent was obtained from participants of this study.

### Magnification system

In addition to the naked eye (control group), Galilean binocular loupe headband systems (Ymarda Optical Instrument Factory, Nanjing, China), which allows a manual adjustment were used at three different magnifications (2.5×, 3.0×, and 3.5×) to perform the restoration procedures.

### Restorative dentistry procedures

Cavity preparations and Class I composite resin restorations were performed on the right maxillary first molar, the left maxillary first molar, the left mandibular first molar, and the right mandibular first molar.

The cavity preparations and restorations were made according to the recommendations of Restorative Dentistry I course of the School of Dentistry of UNESP, Araraquara. MOM-brand dental mannequins with artificial teeth in resin (Manequins Odontológicos Marília (MOM), Marília, São Paulo, Brazil), specific for simulated procedures at the laboratory level, were used in this study. The mannequin was placed on a dental chair to simulate treatment in a clinical setting.

A 1012 round diamond bur (Kg Sorensen–Cotia, São Paulo, Brazil) was used as a rotating instrument at low speed for the cavity preparations. The burs were replaced after every ten preparations (Pazos et al., 2020).

Cavities were restored using a composite resin for posterior teeth (Filtek Z-250 XT–3M), an Almore titanium filling spatula for resin (Duflex®) and a #1 double-ended carver (Millennium—Golgran®).

### Working posture recordings

In order to evaluate the modified CADEP and angular deviation, the working posture adopted during the entire restoration procedures were filmed using three digital cameras (GoPro Hero 4). To standardize and to avoid the error bias of measuring, the cameras were positioned on a leveling tripod, with their optical axis parallel to the floor and perpendicular to the operator, in order to provide side and front views of the operators. The three filming points were established prior to the procedures to allow for adequate visualization of all parts of the body to be evaluated (Pazos et al., 2020).

### Postural assessment

The working posture was assessed using the modified CADEP proposed by Garcia, Wajngarten & Campos (2018) which evaluates students’ compliance with ergonomic posture requirements on a 10-point scale. In this study, the modification of the CADEP corresponds to the non-evaluation of items 1, 2, 3 and 14 considered in the original CADEP. These items are related to the posture of the operator’s legs in the vertical and horizontal directions, with the footrest and with the position of the instruments on the clinical table during the procedure. It was not assessed because they are more related to postural and educational habits of the operator than to the effects of magnification itself.

After watching each complete video, a calibrated researcher (ρ = 1.00) selected the working posture most frequently adopted during the execution of each procedure. Then, each posture was evaluated, with a point being attributed to items considered adequate, half a point for items considered partially adequate and zero for those considered inadequate. At the end, all items were added and could total a maximum of 10 points.

### Angular deviation

From the side view provided by one of the cameras, the images of the lateral angular deviation from the neutral neck position was assessed by a researcher who was duly trained in a pilot study (ρ = 0.83), using the software known as Software para Avaliação Postural, version 0.69 (Laboratory for Biomechanics and Motor Control Federal University of ABC (UFABC), São Bernardo do Campo, São Paulo, Brazil. Available in: http://pesquisa.ufabc.edu.br/bmclab/sapo/) (Ferreira et al., 2010).

### Muscle activation

This study followed the recommendations in the protocol for surface EMG of the non-invasive assessment of muscle (Hermens et al., 2000). Electromyographic signal collection relied on the MyoSystem-BrI portable device (Datahominis Tecnologia, São Paulo, SP, Brazil). A high-performance signal acquisition system and software to control the system, store and process the data were also used. Simple differential active bipolar electrodes were used. The sites consisted of the locations of the muscles under study (right and left sternocleidomastoid muscles, right and left descending trapezius muscles, and right and left ascending trapezius muscles) and the right wrist, the latter of which received the ground wire that served as the reference electrode to ensure signal quality.

The EMG signals were collected continuously, as 120-second cycles, while the simulated restoration procedures were performed, were stored on a computer, and were then processed and analyzed (Milerad et al., 1991). In order to standardize the electromyographic signal, the operators performed resistance-style maximum voluntary contractions for 4 seconds with each muscle group under study (Haddad et al., 2012). The operator then took a 10-min break before beginning the restorations (Pejcić et al., 2016).

The raw EMG data were processed and filtered, and the root mean square values were then calculated with a bandwidth filter from 5 Hz to 5 kHz. The data were then normalized based on the initial maximum voluntary contraction record.

### Statistical analysis

Both for the postural assessment and for the angular deviation of the neck, the examiner’s calibration consisted of the duplicate evaluation of 20 images of each with an interval of 1 week between them. Its reliability was estimated by the intraclass correlation coefficient (ρ). A level of intra-examiner agreement rated at least as “Good” was considered adequate.

A descriptive statistical analysis was performed. After the normality and homoscedasticity assumptions were confirmed, a two-factor analysis of variance (ANOVA) was performed, where one of the factors was “magnification level” and the other “tooth”. Multiple comparisons were performed using Tukey’s post-hoc test or the Games-Howell post-hoc test for each dependent variable in cases of homoscedasticity and heteroscedasticity, respectively. The level of significance adopted was 5%.

In the tables where it was observed statistical significance for teeth and magnification level factors in the different procedures evaluated it was inserted the Total Column/Line to represent the post-hoc test letter.

## Results

Table 1 presents the mean, standard deviation, and summary of the two-factor ANOVA of the final score of CADEP during the cavity preparations and restorations on the first molars, according to magnification level used.

**Table 1:** Mean, standard deviation, and summary of the two-factor ANOVA of the final score of CADEP during the cavity preparations and restorations, according to magnification level used.

Tooth	Magnification level			
	Naked Eye	Galilean 2.5×	Galilean 3.0×	Galilean 3.5×
**Cavity preparation**				
16	8.40 ± 0.26Aa	9.67 ± 0.44Ba	9.97 ± 0.11Ba	10.0 ± 0.00Ba
26	8.62 ± 0.32Aa	9.77 ± 0.41Ba	9.95 ± 0.15Ba	9.97 ± 0.11Ba
36	8.55 ± 0.22Aa	9.32 ± 0.37Bab	9.47 ± 0.25Bb	9.47 ± 0.25Bb
46	8.45 ± 0.32Aa	9.00 ± 0.49Bb	9.45 ± 0.22Bb	9.42 ± 0.29Bb
**Restoration**				
16	8.45 ± 0.15Aa	9.82 ± 0.24Ba	9.85 ± 0.23Ba	9.85 ± 0.28Ba
26	8.40 ± 0.31Aa	9.82 ± 0.29Ba	9.90 ± 0.20Ba	9.90 ± 0.26Ba
36	8.62 ± 0.22Aa	9.32 ± 0.49Bb	9.60 ± 0.35Ba	9.65 ± 0.40Bac
46	8.57 ± 0.29Aa	9.52 ± 0.47Bab	9.72 ± 0.30Ba	9.47 ± 0.34Bbc

**Notes:**

Two-Factor ANOVA (cavity preparations): magnification level (F = 306.432, *p* < 0.001, ηp^2^ = 0.751, π = 1.00), tooth (F = 53.559, *p* < 0.001, ηp^2^ = 0.346, π = 1.00), magnification level *vs* tooth (F = 6.592, *p* < 0.001, ηp^2^ = 0.163, π = 1.00). Games-Howell post-hoc test*; two-factor ANOVA (restorations): magnification level (F = 285.914, *p* < 0.01, ηp^2^ = 0.738, π = 1.00), tooth (F = 9.448, *p* < 0.01, ηp^2^ = 0.085, π = 1.00), magnification level *vs* tooth (F = 5.302, *p* < 0.01, ηp^2^ = 0.136, π = 0.997). Games-Howell post-hoc test*.

* A capital letter for columns; a lower case for lines; the same letter indicates statistical similarity.

In the case of both the cavity preparations and the restorations, the CADEP-scores were significantly lower when working with the naked eye. No statistically significant differences were found between the different levels of loupe magnification. CADEP-scores did not differ significantly between the different teeth involved regardless the procedure. Although, at 2.5× and 3.5× magnification, CADEP-scores were higher in procedures performed in the 16, 26 (upper jaw). Only in cavity preparations, 3.0× magnification produced higher CADEP-scores in procedures in the 16, 26. During restoration procedures there were no significant differences in CADEP-scores regardless the teeth to which the procedure was applied to.

Table 2 presents the mean, standard deviation, and the summary of the two-factor ANOVA of the angular deviation of the neck during the cavity preparations and restorations on the first molars, according to magnification level used.

**Table 2:** Mean, standard deviation, and the summary of the two-factor ANOVA of the angular deviation of the neck during the cavity preparations and restorations, according to magnification level used.

Tooth	Magnification level				
	Naked Eye	Galilean 2.5×	Galilean 3.0×	Galilean 3.5×	Total
**Cavity preparation**					
16	48.84 ± 5.48	40.19 ± 5.02	35.54 ± 5.49	33.40 ± 5.72	39.49 ± 7.99a
26	48.82 ± 5.81	37.21 ± 6.42	33.41 ± 5.09	33.15 ± 5.43	38.15 ± 8.51a
36	43.76 ± 5.67	33.05 ± 5.79	29.27 ± 5.82	30.39 ± 6.74	34.12 ± 8.26b
46	45.60 ± 5.64	30.25 ± 6.71	30.17 ± 5.53	27.94 ± 5.82	33.49 ± 9.18b
TOTAL	46.76 ± 5.96A	35.18 ± 7.04B	32.10 ± 5.95C	31.22 ± 6.25C	36.31 ± 8.84
**Restoration**					
16	56.05 ± 8.27	37.20 ± 5.14	37.17 ± 5.13	38.66 ± 4.99	42.27 ± 9.98a
26	55.42 ± 5.70	39.11 ± 4.90	37.55 ± 4.79	39.92 ± 5.33	43.00 ± 8.87a
36	41.22 ± 9.28	27.04 ± 4.81	27.11 ± 3.40	27.99 ± 6.04	30.84 ± 8.62b
46	40.45 ± 8.12	27.21 ± 4.22	27.36 ± 5.54	25.94 ± 4.34	30.24 ± 8.22b
Total	48.29 ± 10.83A	32.64 ± 7.30B	32.30 ± 6.93B	33.13 ± 8.08B	36.59 ± 10.78

**Notes:**

Two-Factor ANOVA (cavity preparations): magnification level (F = 122.869, *p* < 0.001, ηp^2^ = 0.548, π = 1.00), tooth (F = 20.939, *p* < 0.001, ηp^2^ = 0.171, π = 1.00), magnification level *vs* tooth (F = 1.138, *p* = 0.335, ηp^2^ = 0.033, π = 0.562). Tukey’s post-hoc test*; two-factor ANOVA (restoration): magnification level (F = 143.220, *p* < 0.01, ηp^2^ = 0.586, π = 1.00), tooth (F = 114.952, *p* < 0.01, ηp^2^ = 0.531, π = 1.00), magnification level *vs* tooth (F = 1.076, *p* = 0.380, ηp^2^ = 0.031, π = 0.533), the Games-Howell post-hoc test*.

* A capital letter for columns; a lower case for lines; the same letter indicates statistical similarity.

For both types of procedures performed, the angular deviation of the neck was greater when operators worked with the naked eye, regardless of the tooth involved. For the cavity preparations, the use of 3.0× and 3.5× magnification resulted in an angular deviation less than that seen in cases of 2.5× magnification. The angular deviation of the neck was also statistically significant higher in the preparations and restorations performed in the 16, 26 regardless of the magnification factor of the loupe used. During the restoration procedures, there were no statistically significant differences in operators’ neck angulation between the three different loupe magnifications evaluated.

Table 3 presents the mean, standard deviation, and the summary of the two-factor ANOVA of the EMG normalized values of the right and left sternocleidomastoid muscles during cavity preparations and restoration procedures performed on the first molars, according to the magnification level used.

**Table 3:** Mean, standard deviation and summary of two-factor ANOVA of the EMG values of right and left sternocleidomastoid muscles during cavity preparations and restoration, according to magnification level.

Tooth	Magnification level			
	Naked Eye	Galilean 2.5×	Galilean 3.0×	Galilean 3.5×
**Cavity preparation**				
**Right sternocleidomastoid muscle**				
16	13.66 ± 4.87	11.27 ± 3.75	10.99 ± 4.78	11.37 ± 4.08
26	11.58 ± 4.98	12.62 ± 6.51	13.44 ± 6.95	10.96 ± 3.64
36	10.77 ± 4.83	11.08 ± 3.62	12.23 ± 5.65	13.23 ± 7.57
46	12.48 ± 5.22	12.27 ± 5.20	11.66 ± 4.88	11.54 ± 4.71
**Left sternocleidomastoid muscle**				
16	4.08 ± 3.68	3.74 ± 3.19	4.63 ± 3.63	5.18 ± 3.73
26	5.02 ± 5.13	4.34 ± 3.43	4.68 ± 3.57	4.39 ± 3.75
36	5.16 ± 2.27	4.49 ± 0.99	6.45 ± 3.29	6.67 ± 2.69
46	6.85 ± 2.51	6.54 ± 1.49	7.86 ± 3.35	6.64 ± 1.06
**Restoration**				
**Right sternocleidomastoid muscle**				
16	3.82 ± 1.25	4.57 ± 1.84	4.22 ± 1.87	3.79 ± 1.95
26	4.37 ± 1.56	3.81 ± 2.44	4.15 ± 2.18	4.71 ± 2.02
36	3.59 ± 1.75	3.18 ± 1.21	3.85 ± 1.38	3.49 ± 1.58
46	4.57 ± 1.67	4.45 ± 1.45	3.70 ± 0.88	3.64 ± 1.01
**Left sternocleidomastoid muscle**				
16	2.60 ± 0.88	2.32 ± 1.06	2.57 ± 1.39	2.28 ± 1.14
26	2.41 ± 0.63	2.23 ± 1.12	3.02 ± 1.44	2.66 ± 1.01
36	2.57 ± 0.58	2.53 ± 0.59	2.59 ± 0.50	2.53 ± 0.39
46	2.67 ± 0.47	2.18 ± 1.18	2.21 ± 0.97	2.49 ± 0.75

**Note:**

Two-Factor ANOVA for the right sternocleidomastoid muscle (cavity preparations): magnification level (F = 0.048, *p* = 0.986, ηp^2^ = 0.001, π = 0.058), tooth (F = 0.036, *p* = 0.991, ηp^2^ = 0.001, π = 0.056), magnification level *vs* tooth (F = 0.503, *p* = 0.870, ηp^2^ = 0.030, π = 0.240); Two-Factor ANOVA for the left sternocleidomastoid muscle (cavity preparations): magnification level (F = 0.962, *p* = 0.342, ηp^2^ = 0.021, π = 0.270), tooth (F = 5.678, *p* < 0.01, ηp^2^ = 0.103, π = 0.937), magnification level *vs* tooth (F = 0.309, *p* = 0.971, ηp^2^ = 0.019, π = 0.155); Two-Factor ANOVA for the right sternocleidomastoid muscle (restorations): magnification level (F = 0.079, *p* = 0.971, ηp^2^ = 0.002 π = 0.064), tooth (F = 1.469, *p* = 0.226, ηp^2^ = 0.030, π = 0.383), magnification level *vs* tooth (F = 0.680, *p* = 0.726, ηp^2^ = 0.041, π = 0.326); Two-Factor ANOVA for the left sternocleidomastoid muscle (restorations): magnification level (F = 0.726, *p* = 0.538, ηp^2^ = 0.015, π = 0.202), tooth (F = 0.386, *p* = 0.763, ηp^2^ = 0.008, π = 0.125), magnification level *vs* tooth (F = 0.510, *p* = 0.865, ηp^2^ = 0.031 π = 0.244).

**Figure 1:**
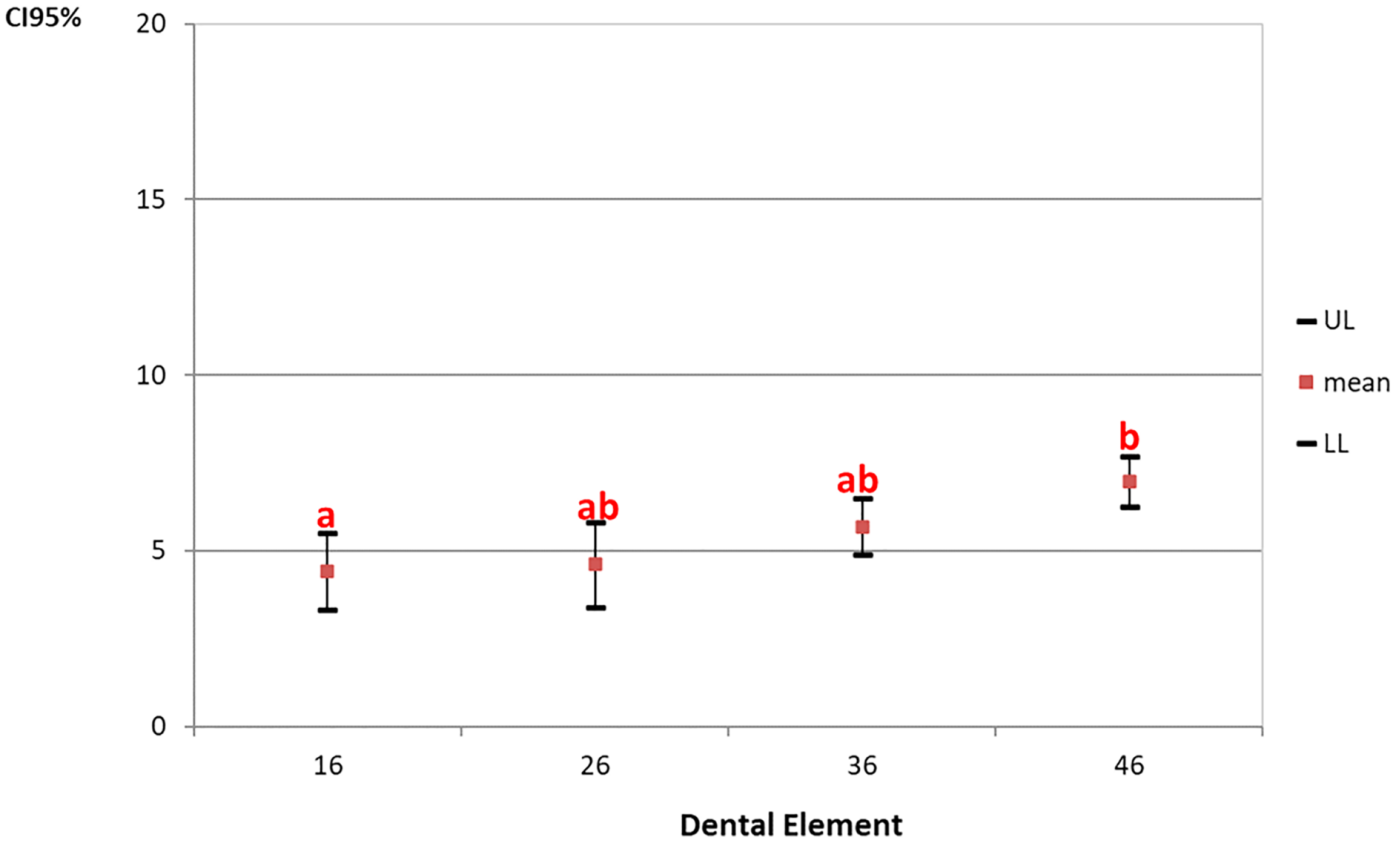
The 95% confidence interval (CI 95%) of left sternocleidomastoid muscle activation organized by tooth being prepared. Games-Howell post-hoc test. a, b-Repeated letters represent statistical similarity.

The left sternocleidomastoid muscle contraction was found to be statistically significant only for the tooth factor during the cavity preparations. Figure 1 presents the 95% confidence interval of left sternocleidomastoid muscle activation organized by tooth being prepared.

Figure 1 shows that the left sternocleidomastoid muscle contraction was found to be lower in procedures performed on the right maxillary first molar and statistically different from the right mandibular first molar, regardless of the magnification level used.

Table 4 presents the mean, standard deviation, and summary of the two-factor ANOVA of the EMG normalized values of the right and left descending trapezius muscles during cavity preparations and restoration procedures performed on the first molars, according to the magnification level used.

**Table 4:** Mean, standard deviation and summary of two-factor ANOVA of EMG values of right and left descending trapezius muscles during cavity preparations and restoration, according to magnification level.

Tooth	Magnification level				
	Naked Eye	Galilean 2.5×	Galilean 3.0×	Galilean 3.5×	TOTAL
**Cavity preparation**					
**Right descending trapezius muscle**					
16	47.54 ± 7.27	48.48 ± 17.71	43.25 ± 11.19	45.44 ± 8.83	46.18 ± 11.65a
26	57.34 ± 10.11	44.64 ± 12.11	46.81 ± 8.47	49.47 ± 11.74	49.56 ± 11.38ab
36	54.92 ± 5.68	43.22 ± 7.98	57.29 ± 12.73	51.47 ± 7.93	51.72 ± 10.16ab
46	56.74 ± 11.37	48.45 ± 9.88	56.27 ± 15.79	55.12 ± 14.58	54.14 ± 13.06b
TOTAL	54.13 ± 9.42A	46.19 ± 12.21BC	50.90 ± 13.33AC	50.38 ± 11.22AC	50.40 ± 11.86
**Left descending trapezius muscle**					
16	80.49 ± 19.89	73.31 ± 20.99	80.89 ± 17.84	83.90 ± 18.45	79.65 ± 18.99a
26	73.69 ± 16.20	57.84 ± 17.77	71.31 ± 19.59	80.02 ± 15.74	70.72 ± 18.61a
36	49.13 ± 14.93	48.32 ± 10.77	54.40 ± 12.68	49.19 ± 14.38	50.26 ± 13.00b
46	61.59 ± 23.97	56.38 ± 20.81	58.79 ± 17.15	56.25 ± 22.47	58.25 ± 20.54b
TOTAL	66.23 ± 21.98	58.96 ± 19.63	66.35 ± 19.46	67.34 ± 22.97	64.72 ± 21.13
**Restoration**					
**Right descending trapezius muscle**					
16	35.28 ± 5.89	38.15 ± 7.11	37.22 ± 7.32	38.45 ± 5.95	37.28 ± 6.45a
26	34.08 ± 4.59	34.50 ± 5.19	34.29 ± 4.84	34.56 ± 4.93	34.36 ± 4.70a
36	28.51 ± 2.71	25.19 ± 2.26	28.01 ± 2.66	25.79 ± 3.76	26.88 ± 3.13b
46	27.45 ± 5.10	26.37 ± 2.85	24.78 ± 2.66	25.13 ± 2.39	25.93 ± 3.46b
TOTAL	31.33 ± 5.69	31.05 ± 7.17	31.07 ± 6.78	30.98 ± 7.19	31.11 ± 6.67
**Left descending trapezius muscle**					
16	31.59 ± 4.85	29.72 ± 5.51	26.18 ± 3.73	25.29 ± 4.12	28.20 ± 5.13a
26	31.86 ± 3.61	30.73 ± 7.52	27.48 ± 4.03	25.23 ± 3.60	28.82 ± 5.47a
36	26.58 ± 3.23	24.55 ± 2.30	24.04 ± 2.60	23.24 ± 2.76	24.60 ± 2.91b
46	27.01 ± 1.63	24.76 ± 3.11	24.29 ± 2.39	24.04 ± 2.32	25.02 ± 2.61b
TOTAL	29.26 ± 4.21A	27.44 ± 5.62AC	25.50 ± 3.45BC	24.45 ± 3.27B	26.67 ± 4.59

**Notes:**

Two-Factor ANOVA for the right descending trapezius muscle (cavity preparations): magnification level (F = 3.336, *p* = 0.021, ηp^2^ = 0.065, π = 0.749), tooth (F = 3.591, *p* = 0.015, ηp^2^ = 0.070 π = 0.783), magnification level *vs* tooth (F = 1.216, *p* = 0.290, ηp^2^ = 0.071, π = 0.581); Tukey’s post-hoc test*. Two-Factor ANOVA for the left descending trapezius muscle (cavity preparations): magnification level (F = 1.838, *p* = 0.143, ηp^2^ = 0.037, π = 0.470), tooth (F = 20.835, *p* < 0.01, ηp^2^ = 0.303, π = 1.00), magnification level *vs* tooth (F = 0.629, *p* = 0.771, ηp^2^ = 0.038, π = 0.301); Tukey’s post-hoc test*. Two-Factor ANOVA for the right descending trapezius muscle (restorations): magnification level (F = 0.042, *p* = 0.989, ηp^2^ = 0.001, π = 0.057), tooth (F = 56.752, *p* < 0.001, ηp^2^ = 0.542, π = 1.000), magnification level *vs* tooth (F = 0.932, *p* = 0.499, ηp^2^ = 0.055, π = 0.450); Games-Howell post-hoc test*. Two-Factor ANOVA for the left descending trapezius muscle (restorations): magnification level (F = 12.224, *p* < 0.001, ηp^2^ = 0.203, π = 1.00), tooth (F = 12.522, *p* < 0.001, ηp^2^ = 0.207, π = 1.000), magnification level *vs* tooth (F = 0.839, *p* = 0.582, ηp^2^ = 0.050, π = 0.404); Games-Howell post-hoc test*.

* A capital letter for columns; a lower case for lines; the same letter indicates statistical similarity.

Considering the cavity preparation, the right descending trapezius muscle showed statistical significance for the “magnification level” (*p* = 0.021) and “tooth” (*p* = 0.015) factors. For the “magnification level” the percentage of right descending trapezius muscle contraction was lower during the cavity preparations at 2.5× magnification. For the “tooth” factor this muscle also exhibited its lowest percentage of contraction when the right maxillary first molar was being prepared. Meanwhile, for the restoration, it was observed statistical significance just for the “tooth” factor, where it was observed greater activity on the right maxillary first molar and the left maxillary first molar than on the other teeth.

Considering the cavity preparation, the left descending trapezius muscle showed statistical significance only for the “tooth” factor (*p* < 0.01), where exhibited greater activity on the upper jaw. For the restoration, this muscle showed statistical significance for the “magnification level” (*p* < 0.01) and “tooth” (*p* < 0.01) factors. For the “magnification level” the activity of this muscle was highest when operators were working with the naked eye. For the “tooth” factor it was observed greater activity on the upper jaw.

Table 5 presents the mean, standard deviation, and two-factor ANOVA of the EMG normalized values of the right and left ascending trapezius muscles during cavity preparations and restoration procedures performed on the first molars, according to the magnification level used.

**Table 5:** Mean, standard deviation and summary of two-factor ANOVA of EMG values of right and left ascending trapezius muscles during cavity preparations and restoration, according to the magnification level.

Tooth	Magnification level				
	Naked Eye	Galilean 2.5×	Galilean 3.0×	Galilean 3.5×	TOTAL
**Cavity preparation**					
**Right ascending trapezius muscle**					
16	16.62 ± 4.11	19.03 ± 6.01	17.03 ± 4.85	20.46 ± 7.64	18.28 ± 5.80a
26	13.85 ± 3.56	14.57 ± 3.53	15.83 ± 5.77	13.79 ± 2.86	14.51 ± 4.00b
36	25.18 ± 11.06	21.19 ± 5.42	24.30 ± 8.14	23.29 ± 6.07	23.49 ± 7.82c
46	26.52 ± 12.42	28.96 ± 11.04	27.91 ± 14.07	28.76 ± 16.28	28.04 ± 13.10c
TOTAL	20.54 ± 10.04	20.94 ± 8.60	21.27 ± 10.00	21.58 ± 10.72	21.08 ± 9.78
**Left ascending trapezius muscle**					
16	16.05 ± 4.19	13.02 ± 3.61	15.87 ± 2.74	14.23 ± 5.54	14.79 ± 4.18a
26	19.36 ± 5.64	18.07 ± 5.56	19.08 ± 5.43	17.09 ± 7.01	18.40 ± 5.78b
36	17.26 ± 3.40	17.61 ± 6.29	17.21 ± 4.59	16.06 ± 5.90	17.03 ± 5.00ab
46	22.87 ± 6.94	25.33 ± 7.38	25.27 ± 10.26	21.60 ± 6.32	23.77 ± 7.73c
TOTAL	18.88 ± 5.66	18.50 ± 7.19	19.36 ± 7.14	17.25 ± 6.58	18.50 ± 6.65
**Restoration**					
**Right ascending trapezius muscle**					
16	13.79 ± 6.30	16.34 ± 4.25	19.17 ± 7.66	16.76 ± 3.91	15.52 ± 5.84ab
26	12.38 ± 4.42	14.57 ± 2.15	14.54 ± 7.47	14.61 ± 2.99	14.03 ± 4.63b
36	17.60 ± 5.68	18.86 ± 5.45	16.55 ± 4.79	17.06 ± 4.84	17.52 ± 5.08a
46	14.23 ± 2.60	16.52 ± 2.29	14.05 ± 3.29	12.98 ± 4.28	14.45 ± 3.35b
TOTAL	14.50 ± 5.14	16.57 ± 3.96	16.08 ± 6.20	15.35 ± 4.25	15.65 ± 4.98
**Left ascending trapezius muscle**					
16	19.23 ± 4.88	16.37 ± 2.48	14.64 ± 3.15	15.44 ± 3.36	16.17 ± 3.94a
26	16.28 ± 3.82	15.08 ± 4.62	16.15 ± 5.30	13.03 ± 4.83	15.74 ± 4.52a
36	13.10 ± 2.99	13.68 ± 3.35	13.25 ± 2.16	14.29 ± 6.08	13.26 ± 3.78b
46	13.69 ± 3.51	15.13 ± 3.56	12.34 ± 1.72	14.31 ± 5.09	13.86 ± 3.68ab
TOTAL	15.58 ± 4.45	15.06 ± 3.58	14.10 ± 3.55	14.31 ± 4.82	14.76 ± 4.14

**Notes:**

Two-Factor ANOVA for the right ascending trapezius muscle (cavity preparations): magnification level (F = 0.105, *p* = 0.957, ηp^2^ = 0.002, π = 0.069), tooth (F = 18.764, *p* < 0.01, ηp^2^ = 0.281, π = 1.00), magnification level *vs* tooth (F = 0.334, *p* = 0.962, ηp^2^ = 0.020, π = 0.165); Games-Howell post-hoc test*. Two-Factor ANOVA for the left ascending trapezius muscle (cavity preparations): magnification level (F = 0.926, *p* = 0.430, ηp^2^ = 0.019, π = 0.250), tooth (F = 16.497, *p* < 0.01, ηp^2^ = 0.256, π = 1.00), magnification level *vs* tooth (F = 0.350, *p* = 0.956, ηp^2^ = 0.021, π = 0.172); Tukey’s post-hoc test*. Two-Factor ANOVA for the right ascending trapezius muscle (restorations): magnification level (F = 1.407, *p* = 0.243, ηp^2^ = 0.028, π = 0.368), tooth (F = 4.797, *p* = 0.003, ηp^2^ = 0.091, π = 0.896), magnification level *vs* tooth (F = 0.862, *p* = 0.561, ηp^2^ = 0.091, π = 0,416); Tukey’s post-hoc test*. Two-Factor ANOVA for the left ascending trapezius muscle (restorations): magnification level (F = 1.177, *p* = 0.321, ηp^2^ = 0.024, π = 0.311), tooth (F = 5.039, *p* = 0.002, ηp^2^ = 0.095, π = 0.911), magnification level *vs* tooth (F = 1.010, *p* = 0.434, ηp^2^ = 0.059, π = 0.487); Tukey’s post-hoc test*.

*A capital letter for columns; a lower case for lines; the same letter indicates statistical similarity.

Considering the cavity preparation, the right ascending trapezius muscle showed statistical significance just for the “tooth” factor (*p* < 0.01), exhibiting greater activity on the lower jaw. During the restorations, this muscle showed statistical significance just for the “tooth” factor (*p* = 0.003) presenting lower activity on the left maxillary first molar and the right mandibular first molar.

Considering the cavity preparation, the left ascending trapezius muscle showed statistical significance only for the “tooth” factor (*p* < 0.01), presenting greatest activity on right mandibular first molar. During the restorations, this muscle showed statistical significance just for the “tooth” factor (*p* = 0.002) presenting lower activity on the left mandibular first molar.

## Discussion

The objective of this study was to determine the effect of different levels of Galilean loupe magnification on working posture as measured by the modified CADEP, angular deviation from the neutral position of the neck, and muscle activation in the neck and upper back region during restoration procedures under simulated clinical conditions. The use of Galilean loupes at all magnification levels during the cavity preparations and restorations was found to positively influence working posture as measured by the compliance with ergonomic posture requirements and neck angulation.

The improvement in working posture result from the use of magnification systems may be explained by the improved visualization of the operating field (Carpentier et al., 2019; Branson et al., 2004; Branson et al., 2018; García-Vidal et al., 2019). Another issue to consider is the focal distance required by magnification systems, which controls the distance between the operator’s eyes and the patient’s mouth and thus prevent the operator from positioning themselves at an improper distance from the operating field, allowing them to adopt a more ergonomic posture.

In other studies, similar results have been reported. In a study that evaluated the effect of magnification on dental students’ working posture during the preclinical training phase, magnification was found to have a positive impact on posture and to reduce operators’ risk of developing musculoskeletal disorders (Kamal et al., 2020). Other studies have observed better posture scores among students who used loupes to perform periodontal procedures when compared to students who worked with the naked eye (Maillet et al., 2008; Branson et al., 2004). Another issue to consider is the combination of magnification loupes and an ergonomic stool that was found to have a positive influence on dental students’ working posture (Dable et al., 2014).

In studies that have considered the angular deviation of the neck, magnification was found to enable improved neck angulation during the preclinical training phase and during clinical procedures such as periodontal probing and diagnostics (Wajngarten & Garcia, 2019; Kamal et al., 2020; Branson et al., 2018). Although the magnification system has provided a reduction in the angular deviation of the neck, it still remains higher than what is considered neutral. This may be because, according to Rucker et al. (1999), the declination angle of the magnification loupes’ lenses must be finely adjusted for the operator to achieve a balance between eye stress and neck angulation. In this study, fully adjustable head-band loupes were used, which allowed the change of each of their characteristics, including the declination angle, each time the loupes were used. As this is a very delicate adjustment, the magnification loupe not specially adjusted for the operator in question may have caused greater neck angulations.

Another issue to be considered is the relationship between tooth location and angular deviation of the neck. It was observed that the angular deviation of the neck for both cavity preparations and restorations was greater during the work on the upper arch (Table 2). This could be due to the declination angle adjustment, which may differ on both arcs. It can be suggested that this angle has not been properly adjusted for the upper arch, resulting in a greater downward tilt of the head to be able to visualize the teeth in this arch (Rucker et al., 1999).

Regarding muscle activity in the neck and upper back, it was found in this study that the use of these loupes did not affect muscle activation. These results differed from those reported in prior research. Other authors reported less muscle contraction in the upper back muscles during the use of magnification loupes to perform preclinical activities (García-Vidal et al., 2019; López-Nicolás et al., 2019). One reason for this difference between this study and others may be the type of lenses selected. In this study, only headband loupes were used; other studies have used through-the-lens loupes. Headband loupes tend to be heavier, and the extra weight may cause greater muscle contraction, especially for inexperienced users. It is important to note that an increase in muscle activation during loupe use could support a contraindication for loupe use. The results presented in this study regarding muscle tension differ from what has been found in previous literature, therefor further studies about implementation of magnification in education environment should be conducted.

Tooth location was also found to influence the extent of muscle activation. The sternocleidomastoid muscle showed greater contraction for cavity preparation on right mandibular first molar, which may be related to one of the functions of this muscle, to allow the anterior flexion of the neck. The descending trapezius muscle presented greater contraction during the restorative procedures in the upper arch. This muscle acts in the elevation of the head, therefore, its greater activity can be justified by the need to keep the head forward.

Overall, it is possible that the greater muscle activity for cavity preparation in the lower arch is not related to the difficulty of visualization but to the access of the teeth, resulting in greater muscle load for movement control. On the other hand, it can be assumed that the greater muscle activity in the upper arch is related to the greater need to visualize restorative procedure details and not to movement control, as it is a reversible procedure. Other study in the literature has reported worse working posture during cavity preparations performed on the lower dental arch (Kamal et al., 2020). To our knowledge, however, no prior studies have considered the effect of tooth location on the operator’s muscle contraction.

The results presented here therefore support previous recommendations for the use of magnifying loupes that may help prevent the development of musculoskeletal disorders by improving practitioners’ work posture (Wajngarten & Garcia, 2019; Branson et al., 2004; Alhazzazi et al., 2016). However, the long-term effect of the magnification loupes on the working posture and the eye muscle fatigue should be considered before implementing these devices in the educational environment.

The study design proposed for this manuscript was laboratorial, conducted in a standardized condition by simulating the performance of clinical procedures in order to control possible interference caused by the difficulties faced when taking care of a real patient. Although it may be a limitation, due to the scarcity of studies with this approach, it is believed that it can contribute to the area of Occupational Health and to serve as a reference for other studies including the treatment of real patients.

## Conclusions

It can be concluded that the use of Galilean magnification loupes while performing preclinical restorative dentistry procedures had a positive effect on compliance with ergonomic posture and the angulation of the neck; whilst, these loupes did not influence muscle activation in the neck or upper back region.

## Supplemental Information

10.7717/peerj.13021/supp-1 Supplemental Information 1 EMG, CADEP and angular deviation of neck measurements for cavity preparation and restoration.

## Additional Information and Declarations

### Data Availability

The following information was supplied regarding data availability: The raw data are available in the Supplementary File.
